# Inverted follicular keratosis of the lip

**DOI:** 10.11604/pamj.2014.18.304.4984

**Published:** 2014-08-15

**Authors:** Mariem Mohamed

**Affiliations:** 1 Department of Dermatology, Monastir University Hospital, Monastir, Tunisia

**Keywords:** Follicular keratosis, lip, benign tumor

## Image in medicine

Inverted follicular keratosis is a benign tumor of the skin that is believed to arise from the infundibular portion of the hair follicle above the level of entrance of the sebaceous duct. It was described originally by Helwig in 1954 and since then a number of case reports have been reported in the dermatology and pathology literature. A 55-year-old woman was referred for evaluation of a keratotic lesion on the upper lip. The lesion had been noticed 12 weeks previously, and was asymptomatic. The patient was otherwise well. On examination, a pedunculated nodule, 8-12 mm in diameter, with a keratotic surface, was present over the upper lip midline skin. On histological examination, the lesion showed a papillomatous surface, with the formation of a central mass that invaginates into the connective tissue. The epithelium is sharply circumscribed and forms a cupshaped mass. The tumor mass was composed of a proliferation of basaloid cells, and concentric swirls of spinous cells called squamous eddies. There are no mitotic figures. The pathological diagnosis was of an inverted follicular keratosis.

**Figure 1:**
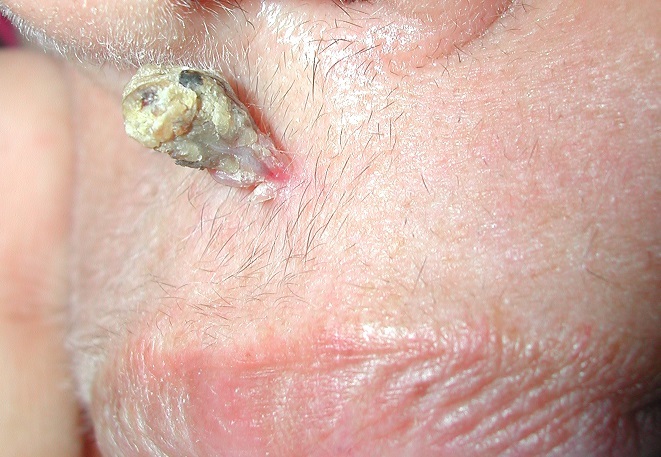
Pedunculated nodule, 8-12 mm in diameter, with a keratotic surface, in the upper lip midline skin

